# An Assessment of Urban Park Access Using House-Level Data in Urban China: Through the Lens of Social Equity

**DOI:** 10.3390/ijerph17072349

**Published:** 2020-03-31

**Authors:** Siqi Yu, Xigang Zhu, Qian He

**Affiliations:** 1 School of Architecture and Urban Planning, Nanjing University, Nanjing 210093, China; zhuxigang522@hotmail.com; 2 College of Architecture, Planning, and Public Affairs, University of Texas at Arlington, TX 76019, USA; qian.he@mavs.uta.edu

**Keywords:** public park, green space, accessibility, spatial differentiation, social justice, environmental equity

## Abstract

The various benefits of urban green space are gaining increasing attention nowadays. Hence, the distribution of green space has become a scrutinized concern for spatial equity among local governments and the planning scholars. This study is the first quantitative evaluation of urban park accessibility using house-level data in urban China, from the perspective of social equity. We chose Nanjing as the empirical case and examined 2709 real estate units and 79 parks within the city. Accessibility is measured by the 10-min walking distance from homes to the adjacent urban parks. Using the Street Network Analysis model in ArcGIS and the statistical methods in SPSS, the result shows that 60.5% of the real estates in Nanjing are located within a 10-min walk to access urban parks. However, this accessibility is positively correlated with housing prices, and negatively correlated with the age of the buildings, holding all other factors constant. While affluent homeowners capture a high-quality green amenity, newly-built low-income communities, where most residents are classified as a vulnerable population, have the lowest percentage of accessible green space. This study reveals the existing spatial disparities of urban park accessibility among different socio-economic groups in Nanjing, China. Additionally, we found that urban redevelopment projects with greening and the large-scale affordable housing construction are pricing out the urban poor and rural immigrants from the inner city to the urban peripheral areas. This will reduce the accessibility to urban parks and other public service facilities among the lower income families, and exacerbate the inequality among the rich and the poor in terms of their quality of life. Main findings of this study can inform policy decisions regarding equitable park provision in the construction of the green city and the sustainable development in urban China and other developing countries.

## 1. Introduction

Green space, including any kind of vegetation covers such as parks, urban forest, gardens, greenways, and tree canopy covers is not only a key ecological factor but also provides various benefits in health, economy, society, and environment to alleviate some of the negative effects of urbanization [1,2,3,4,5,6]. For instance, green space can improve air quality [1] and reduce flooding risk, heat island effect, and greenhouse gas emission [2]; it provides habitat that supports biodiversity [3] and encourages residents to participate in outdoor sports [4], reducing the rates of disease associated with air pollution [5]; high-quality green space strengthens the identity of an area, making it a more attractive work and living space, and thereby enhance local economic potential and land use value [6]. 

The increasing attention on green space benefits raises a question for urban sustainable development: With the support of public funding, are urban parks always equitably distributed within cities? To address this issue, there are a considerable amount of studies about the distribution of the urban park among different social groups based on income, age, gender, racial characteristics, political power, and other axes [7,8,9,10,11,12,13,14,15]. In this vein, compared with the white and wealthy, minority races and low-income groups are less likely to receive urban parks or recreational projects in their communities [7,8,9]. Nevertheless, some studies contend that public facilities such as urban parks are evenly distributed in which disadvantaged groups gather [10,11,12,13]. One possible explanation for these different outcomes could be the inconsistency of evaluation metrics. The local variations in the measure of accessibility, such as park proximity, acres of parks, and park quality could impact the outcome of the calculation [14] and the priority of these metrics varies across different socio-economic groups [15]. Moreover, traditional aggregated data and the unit of analysis, like zip codes, may be inadequate in the evaluation of social equity, especially for highly localized services, for example, parks, gardens, and playgrounds. Therefore, Hewko suggested that finer resolution data like house level should be integrated to better indicate the spatial distribution of individuals [16]. 

In the context of Chinese cities, social inequality has become one of the most scrutinized issues, even though the accessibility of public facilities and spatial equity are new concepts. In fact, studies suggest that China has transformed from relative egalitarianism into a society characterized by a widening income gap since the reform of the socialist market economy [17]. Continued socio-economic inequality has also led to housing segregation along with rising housing values and deteriorated housing affordability [18]. After the 1998 housing reform characterized by privatization and marketization, large-scale and high-frequency replacement of residential population occurs under the filtering mechanism of housing prices. Residents with the similar consumption capacity and stratum traits can gather in specific urban spaces to form homogeneous neighborhoods, while further different socio-economic groups are isolated from each other [19,20,21,22,23,24,25]. Consequently, the growing spatial segregation between the rich and the poor has intuitively raised concerns about whether public resources, such as urban parks, are equitably available among multiple socio-economic statuses. To date, there is little empirical evidence on the assessment of the urban park’s accessibility from a spatial equity perspective, even though environmental justice is closely related to the goals of China’s sustainable development policy [15]. Among the existing studies, most are limited by the aggregated data at a relatively coarse scale, such as the “Jiedao” (sub-district) level, and fail to capture the micro-scale issues among the different social groups [13,26,27]. In addition, their data on the socio-economic attributes of the residents originate from the National Census in China. However, the latest National Census was conducted in 2010, which would be hard to reflect current urban socio-spatial structure [13,27].

This article is the first quantitative evaluation of urban public parks’ accessibility among the multiple socio-economic groups in the context of residential segregation in urban China. We chose Nanjing as the empirical case and evaluated the accessibility to urban parks using the 10-min walk from home as the threshold. With the aid of the Street Network Analysis model in ArcGIS and the SPSS statistical analysis method, taking housing attributes as indicators for socio-economic characteristics, our study then illustrated the spatial distribution of different groups and urban parks from the perspective of social equity. The result of this study provides important implications for municipal decision-making in the improvement of the built environment and the allocation of urban services and amenities. It can also inform policy decisions regarding promoting social justice and sustainable development in China, especially among the current era of Ecological Civilization.

This paper is structured as follows: Part two is the existing literature on the assessment of social equity regarding access to green space. We further analyze the major discussions on social inequality issues in urban China to develop our theoretical framework. The third section explains the methodology used in this study and our data sources. Part four presents the analysis and results, and the last section summarizes the main findings than can inform policy decisions regarding equitable park provision.

## 2. Literature Review

With the increasing attention to practical policymaking, the issue of equal access to public services or amenities has then become of great importance for both planning scholars and local governments, comprising parks, playgrounds, public transportation, and food outlets. Among these amenities, urban park is a vital element of sustainable landscape with its environmental, social, and economic benefits to the urban area [1,2,3,4,5,6]. Moreover, as one type of public goods supported by public funding, urban parks are the key target for these researches of equal access. From the perspective of social equity and environmental justice, researches emphasize whether public services are distributed in an equal pattern among multiple socio-economic groups. This “equity mapping” reflects the relationship between the spatial distribution of public services and the residential location of different socio-economic groups, especially the elderly, minority races, low-income households, and the disadvantaged population who are in need of better access [8]. 

A few articles and reports reviewed the association between the distribution of parks and vulnerable groups [7,8,9,10,11,12,13,14,15]. Through one of the most comprehensive studies, the National Recreation and Park Association notes that there is consistent evidence of disproportion in park provision in the USA, where park services in low-income communities with people of color are significantly lower than in the white and wealthy groups [9]. However, some research contend that there is an impartial distribution of public facilities such as urban parks in areas where disadvantaged groups gather. As detailed in Macintyre’s study, disadvantaged neighborhoods are in fact favored in terms of accessibility to health-promotion facilities compared to other groups [10]. Using empirical evidence from Chicago, Mladenka’s study highlights that race is not a determining factor in the distribution of parks, however, the social class may be [11]. On the contrary, Koehler indicates that the determinants of public facilities are more exposed to professional decision-making processes [12]. 

One possible explanation for this difference could be the inconsistency of evaluation metrics. The measure of park proximity, acres of parks, and park quality could impact the outcome of the evaluation [14]; additionally, the priority of these metrics varies across different socio-economic groups [15]. Some studies evaluate whether the distance to the nearest park or playground is related to demographic variables [7,28,29] and how much difference of the land value there is between within and outside of a quarter-mile buffer from a park [30,31]. However, park proximity describes the distance from a residential location to the nearest park, which mainly reveals the possibility of walking to the park, regardless of public access influenced by the size and quality of the park. Rigolon further studies 49 empirical researches and thought in terms of the access to parks there were inconclusive findings for proximity, but striking imparity for acreage and quality [14]. 

Moreover, geographic scale is an integral part of these researches and practices in urban spatial equity. In a growing body of studies, measuring spatial distribution based on various scales or geographical partitions can lead to different results [7,8,9,10,11,12,13,14,15,32,33,34]. Most spatial equity researches are still limited to the use of large-scale aggregate data such as zip code that often fail to capture the micro-scale issues of social groups and communities. Aggregate data may be particularly inadequate when the evaluation involves highly localized services, such as parks, gardens, and playgrounds. Therefore, Hewko suggests that future research should integrate finer resolution data to better present the spatial distribution of individuals in the neighborhood, thereby reducing clustering errors [16].

In many western countries, geo-referenced data on urban infrastructure services, such as road networks and house locations, is available to accurately estimate the origin-to-destination distance at house levels nowadays [32]. Besides, some housing surveys include various characteristics, which are related to the socio-economic status of households. The data of the real estate market containing the social attributes of the residents can reflect the social space structure and evolving trend of the city so that the spatial relationship between households and urban services can be estimated [19,20,21,22,23,24,25]. Hence, it is possible to improve the accuracy and reliability of the spatial equity assessment of urban amenities and services.

In China’s context, although the accessibility of public facilities and spatial equity are both new concepts, it is no doubt that social inequality has become one of the most scrutinized fields since the reform of the socialist market economy. In fact, studies suggest that after economic reform in 1979, China’s market has transformed from relative egalitarianism into a society characterized by a widening income gap [17]. The current researches on inequality mainly focus on the income gap between different social classes [35], housing inequality [18], the uneven supply of facilities in different regions [36], and the unequal level of individual social achievements [37]. 

There are abundant researches indicating that there is residential socio-spatial differentiation and that the choice of urban housing could reflect the socio-demographic status for the residents in urban China [19,20,21,22,23,24,25]. Housing prices not only reflect the features of urban spatial structure and residential spatial segregation [19] but can also draw the mapping of disparities in economic status and class attributes, among multiple purchasing groups [20]. The differences among the growth of urban housing prices also affect the social wealth distribution and the choice of housing locations for potential buyers and further promote the re-differentiation of the urban socio-economic groups [21]. In other words, the consumption of housing could represent class-structuration in urban China, and the differentiation of housing prices, or the spatial heterogeneity of urban houses, actually reflects socio-spatial inequality [22,23]. As a result, the data of a secondary market of housing prices are often used by some scholars to demonstrate the structure of urban residential space [24,25]. For example, Song (2010) used the housing price as an indicator to describe the gentrification process and residential differentiation in Nanjing [25].

A crucial component of social equality research is the needs of marginalized groups. Within the vulnerable groups in urban China, existing literature mainly identified two types of the new urban poor: The laid-off workers and rural migrants [13,38,39]. The laid-off workers lack enough skills and education, thus it is very hard for them to find new jobs or to leave the poor areas [38]. Meanwhile, the rural migrants are more likely to engage in low-paid and dangerous jobs compared to the local residents [39]. Moreover, continued socio-economic inequality led to housing segregation, due to the low affordability of the new urban poor [18]. The consequence of the residential segregation is that the laid-off workers and rural migrants are more likely to live in poor areas, which further increases the possibility of poverty [38]. 

To date, there is little empirical evidence on the assessment of the urban park’s accessibility from a spatial equity perspective, even though environmental justice is closely related to the goals of China’s sustainable development policy [15]. Using data from 285 prefecture cities across the nation in China, Chen reveals a negative correlation between economic development and urban public green space, which indicates that access to urban green space in China’s megacities is worsening [26]. Some researchers note spatial mismatch exists between the distribution of urban public parks and that of socially vulnerable groups in Changting [27]. Nevertheless, Xiao chooses Shanghai as the case study city and finds that vulnerable groups are favored over more affluent citizens regarding access to urban parks [13]. These studies, however, ignore the population distribution pattern or assume that different groups are evenly distributed in statistical units, which to a certain extent affects the evaluation results. This shortage is directly related to the application of aggregated data. To address this modifiable areal unit problem, Yin introduced an accessibility evaluation on the house scale in Chinese cities for the first time [40]. However, due to the lack of information on housing attributes, there is no direct correspondence analysis in his study. 

Currently, the question of whether different social groups can use urban parks fairly is still waiting to be answered. In a densely populated environment like urban China, it is rarely known whether marginalized groups are having a lower quality of public services, given that China has always lacked green space and the quality of services provided to the entire population is rather low [13]. Therefore, it is extremely important to incorporate an equity-oriented approach to assess the urban parks in Chinese urban settings.

## 3. Methodology

### 3.1. Study Area

This paper took Nanjing (31°14′–32°37′ N, 118°22′–119°14′ E) as the study area. It is one of the most populous and developed cities in eastern China. In 2018, the population of Nanjing was 8,430,000, and the urban built-up areas were 817 km^2^ [41]. This megacity is surrounded by mountains and rivers, such as Xuanwu Lake, Qinhuai River, and Zijin Mountain, and has abundant green resources. With the rapid expansion of urban land, Nanjing municipal government has supported increasing green space as part of its urban green infrastructure planning efforts launched in 2013, making the green coverage rate reaching 45% in 2018 [41]. Specifically, the green space of the metropolitan area increased from 8250 hectares in 2010 to 35,756 hectares in 2018 while urban park area increased from 1725 hectares to 7207 hectares according to the Nanjing Statistical Yearbook (2010–2018). 

**Figure 1:**
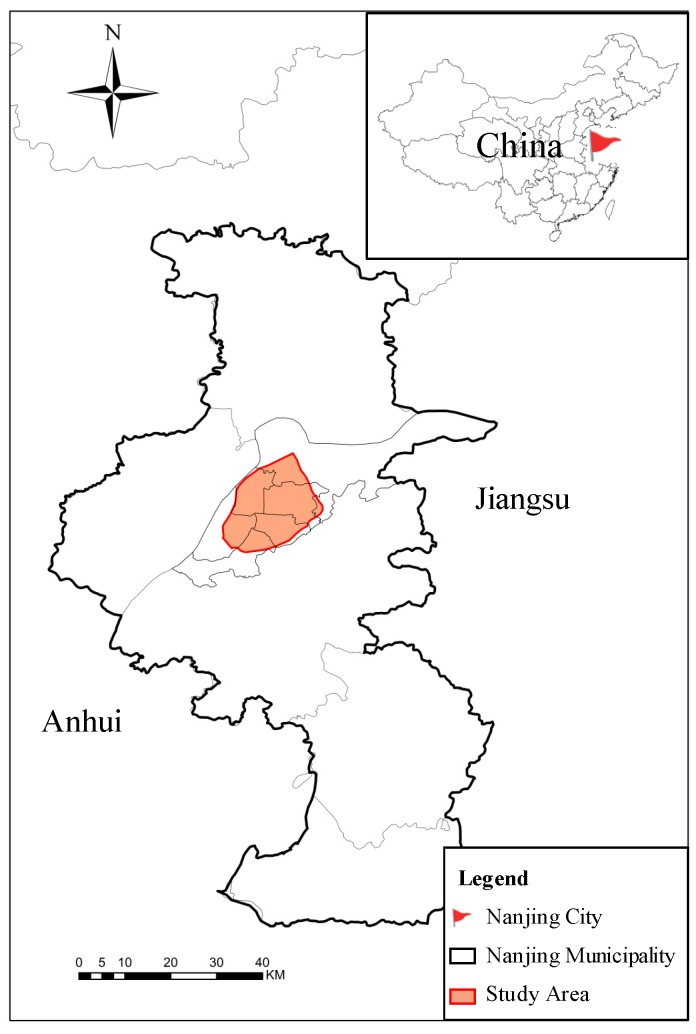
Location of Nanjing, China.

Our study focuses on the main urban area with a high population concentration and a long history of development, which is enclosed by the external ring road (Figure 1). This area comprises six administrative districts: Gulou, Xuanwu, Qinhuai, Jianye, Yuhuatai, and Qixia. Among them, the area enclosed by the city wall of the Ming Dynasty is called the inner city. Before the 21st century, this inner area of 41 km^2^ was once inhabited by about 1.5 million people and was the main container of Nanjing’s population, culture, business, education, and medical services [25].

### 3.2. Data Source

**Figure 2:**
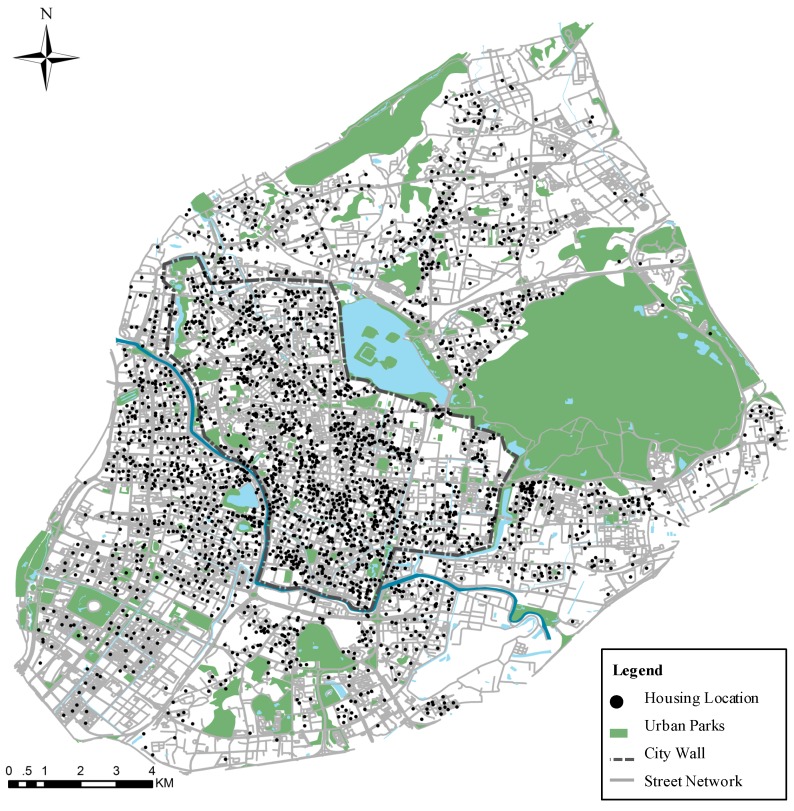
The distribution of parks and housing units in the study area.

In our study, two categories of spatial statistics were required to conduct the analysis upon accessibility to urban parks on the house level, namely park and housing data. For the park data, a total of 79 parks in the central area of Nanjing were included Figure 2. We used the Autonavi electronic navigation map (AMAP) to extract the parks, which contained the latest data and fine granularity [42]. AMAP is one of China’s most popular navigation maps and its data come from commercial remote sensing satellites. Then, we merged the same park for those where the extents were interrupted by a road and assigned the attributes of parks by manual identification. Considering the function of parks is based on service quality with a certain threshold, we then check the consistency between the parks that we extracted and the ones listed on the current parks by the official. Specifically, these selected parks in our study all are included in the “Plan of Urban Green Space System, Nanjing City (2017–2035)” which is officially published by Nanjing Municipality in 2018 [41]. Consequently, unnamed green spaces along the street with isolation functions were not considered in this study. Furthermore, private green areas such as in the gated communities and golf clubs were also not included. Finally, we used spatial modeling methods including GIS and remote sensing technology and selected 79 city parks which covered a total area of 250 km^2^ for this research.

Housing data were collected in December 2018 from the SOFANG website [43]. Since large-scale aggregate data failed to reflect the socio-economic status of residents under the rapid urbanization in China, the evidence of a housing market would be used to describe the socio-spatial structure to improve the accuracy and reliability of the spatial equity assessment [19,20,21,22,23,24,25]. These data of real estate properties were pre-processed to remove any abnormal values and ultimately obtained 2609 records representative of the housing in Nanjing (Figure 2). Their attributes contain the name of real estate, housing price in the second-hand transaction market, the administrative division, age of the building, Home Owner Association fee (HOA fee), community size (number of dwelling buildings in the community), and coordinates of latitude and longitude. Table 1 summarizes all the variables used in this study and general descriptive statistics, with a total of 2609 samples. 

At last, the present land-use map and street-level road network map of Nanjing (2018) were corrected by combining the data from the fieldwork through the manual inspection. Through digital processing and spatial coordinate system calibration, data features including the parks, houses, rivers, and roads were then converted into GIS vector data to establish the integrated GIS database for further accessibility examination.

### 3.3. Network Analysis Method

#### 3.3.1. Measure Method of Accessibility

In this descriptive research, we calculated the accessibility of urban parks using the network analysis method. Accessibility refers to the quantitative expression of the desire and ability of residents to overcome obstacles such as distance, travel time, and expenses to reach a service facility or activity venue [7]. Since the 1950s, accessibility analysis has been widely used to study the rationality of the spatial layout of public facilities such as urban green space [7,16,31,33,44]. The accessibility has been evaluated by many scholars through a variety of methods, mainly including buffer zone, minimum distance, gravity index, and travel cost [7,44]. The calculation of the minimum distance method is relatively simple, and it has been widely used in empirical studies. However, its limitations are also obvious because the use of Euclidean distance differs from the travel routes of residents in reality. The calculation of the gravity model method is more complicated, considering the impact of the metrics varies on urban park access such as park proximity, acres of parks, and park quality, but the Euclidean distance is still used to take a measure. The travel cost method is complicated to calculate. In this method, the network distance is used to minimize the total cost of travel between origin and destination, which is close to the actual travel distance of the residents. With the rapid development of computer hardware and the improvement of GIS functions, the travel distance method is showing great potential in the accessibility evaluation of urban parks. Hence, we measured the distance cost that people spent to reach nearby parks by walking with the aid of the network analysis in ArcGIS platform.

#### 3.3.2. Standards of Accessibility Level

Considering residents are unwilling to access public urban parks that require a long travel journey, the threshold distance on accessing urban parks is of great concern [28]. Therefore, the level of urban park access can be divided based on the time cost to obtain park green space services. The U.S. Department of Transportation’s 2012 National Survey of Bicyclist and Pedestrian Attitudes and Behavior shows that 61% of residents surveyed are willing to walk for an average of 1.3 miles for exercise, leisure, entertainment, and walking their dogs, which is equivalent to the 10-min round-trip walk to a park located a half-mile from home [45]. The 10-min walk from home is also used as the evaluation standard for urban park access in the U.S. Park Score Index [46]. Since China does not have a survey of this type at the national level, drawing on existing research and practices in the United States, a 10-min walk is selected as an accessibility indicator, and the service of urban parks is divided into five levels as shown in Table 2.

#### 3.3.3. Technology Route

As for the technology route, the process of accessibility evaluation is specifically as follows: Firstly, create a network dataset accurate to a street feature in ArcGIS; secondly, calculate the intersection of urban park and streets, and determine them as the park entrances. The official park entrances may not cover all the paths through which people get into the park due to the fact that many parks are not gated in China. Therefore, we adopt the intersection of the street and urban parks to define the entrance of the park rather than use the official entrance in order to assure the validity; thirdly, use the ArcGIS network analysis to calculate the time from the house to the nearest park. We use the gate of the community as the entrance for the gated communities. Meanwhile, for the open community, we chose the intersection of the street and the community boundary as the entrance; at last, classify the accessibility levels according to the time cost of the nearest park green space service obtained by the residential community.

## 4. Result and Discussion

### 4.1. Results of Park Access Assessment

Table 3 shows the statistics results of the percentage within a 10-min walk to park in the 2609 real estates in Nanjing. In general, 60.44% of Nanjing’s real estates live within a 10-min walk of a park. A large proportion of residents’ units (42.32%) can enter a park within 5–10 min’ walk of home. Only 11% of real estates take more than 15 min to a park. In the inner city, the percent of real estates within a 10-min walk to park is 72.37%, while the suburb accounts for 49.86%. In particular, no residents of the inner city could access a park for more than 15 min.

To compare these accessibility levels in Nanjing, we introduce the Park Score index in the United States. Developed by the Trust for Public Land, Park Score is a comprehensive index that describes the quality of urban park systems and the component of park access in this index is frequently used as an indicator for spatial equity in many academic studies [47,48,49,50]. According to the Park Score index, in the 100 largest cities in America, the range of accessibility within the 10-min walk of home is from 28% to 100%, with a median of 65%. In contrast, the level of accessibility in Nanjing is very nearly the same as the national media in the U.S. Among them, the accessibility of the inner city is even higher than the average level, but in the meantime, the result in the suburbs is slightly lower. Consequently, most residents in Nanjing can conveniently enjoy the benefits of green space and the inner-city residents have higher access to urban parks.

**Figure 3:**
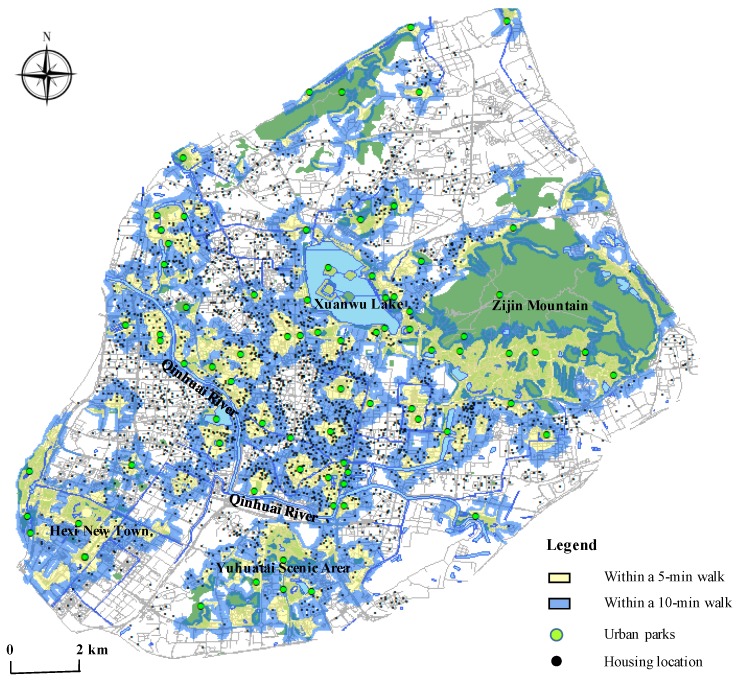
The evaluation of Accessibility among 79 public parks in Nanjing.

Figure 3 is the spatial distribution map of accessibility evaluation of 79 public parks. From the perspective of urban spatial structure, the areas with high accessibility are mainly concentrated in “one belt, four zones” in Nanjing: The one belt refers to the Qinhuai River, and the four zones include Zijin Mountain, Xuanwu Lake, Yuhuatai Scenic Area, and Hexi New Town. As a form of public investment, these areas with high urban park access are significantly exposed to local municipal endeavors. As a result of urban river restoration projects led by the municipal government over many years, a series of riverside parks appear along the Qinhuai River, the mother river of Nanjing. This urban green belt not only benefits nearby residents tremendously, but Nanjing Municipal Government also won the “Special Habitat Honor” which is the first time an official agency in China was awarded by the United Nations. Xuanwu Lake, Zijin Mountain, and Yuhuatai Scenic Area are well-known historical parks with a long history under the management of the Nanjing Municipal Bureau of Parks as a crucial part of the urban green infrastructure system. Promoted and maintained by mega-events, including the 10th National Games of 2005 and the 2010 Nanjing Youth Olympic Games, Hexi New Town is a newly-built urban area after 2000 and intends to be a modern landmark in Nanjing [51]. With the state-sponsored starting point, after the bureaucratic decision-making process, the landscape of this district is elaborately designed with various green spaces and makes residents high access to urban parks.

### 4.2. Correlation between Housing Attributes and Accessibility

In this part, we examine the correlation between housing attributes and accessibility in Nanjing. First, the diagnostic test for collinearity is performed to mitigate potential biases in advance. The four attributes of housing data contain housing price, age of the building, HOA fee, and community size. The variance inflation factors (VIF) for all variables of housing attributes range from 3.21 to 5.85, less than 8 (a suggested cut-off number for multicollinearity), indicating that no serious multicollinearity could be detected amongst all independent variables [52]. Second, the accessibility level and the housing characteristics are normalized by using a range standardization method. Based on the SPSS platform, we then applied the correlation analysis with Spearman’s rank correlation coefficient and Kendall’s rank correlation coefficient to analyze the relationships between housing attributes and accessibility in the 2609 real estates of Nanjing. And the correlation test uses a two-sided test. The result is shown in Table 4.

Through the sequencing variable correlation analysis, firstly, there is a positive correlation between the accessibility level and the housing price, significant at the 0.01 level. There is a tendency to improve in accessibility, as residential housing prices rise. That is, the residents living in luxury real estates enjoy better green space services. Secondly, the age of the building is significantly negatively correlated to the level of accessibility. The older the residential community, the higher the accessibility of urban parks, revealing that urban residents in the old areas have better access to urban parks than the newly-built districts. Finally, regarding the HOA fee and community size, there is no significant correlation between these two variables.

### 4.3. Unequal Residential Distributions and Park Access

For a country with the implementation of housing privatization policy, evidence from the housing markets can help describe the socio-spatial prospects instead, due to circumstances lacking statistics on the urban population [25,53]. After the 1998 housing reform characterized by privatization and marketization, large-scale and high-frequency replacement of residential population occurs under the filtering mechanism of housing prices in urban China. The proportion of private housing in Chinese cities and towns was up to 96% nowadays. This article hereby used housing data from the secondary market as indicators for the distribution of social groups. For the purpose to analyze the spatial pattern between social groups and park access, we applied geostatistical interpolation techniques into housing price and age of the building respectively to quantify the spatial autocorrelation, and then overlay the park access distribution onto the mapped spatial distributions of two variables. 

**Figure 4:**
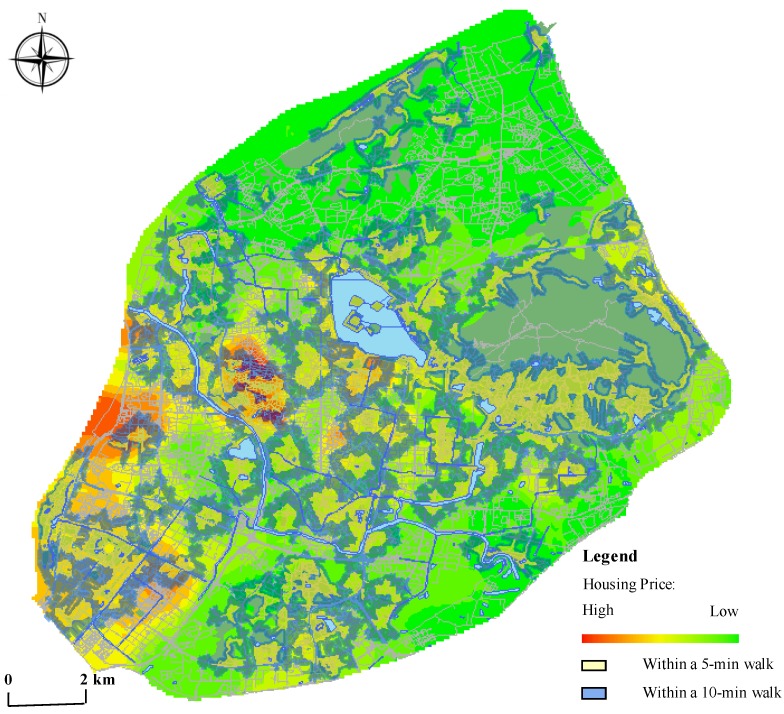
The distribution of park Accessibility and the housing prices in Nanjing.

The overlay analysis of the accessibility and housing price distribution in Nanjing (Figure 4) illustrates that there exists a spatial disparity of green space service in social groups under different living conditions. The areas with higher accessibility are coincident with the high price points, and the housing prices are comparatively lower in the areas that are not covered by the 10-min walk from home to urban parks. For example, in the inner city, high housing prices appeared near Xuanwu Lake and Zijin Mountain. Adjacent to the natural resources, these communities enjoy a high-quality landscape and were referred to as one of the wealthy communities where privileged residents gather in the early time. Another point of high housing price in the suburb is the Hexi New Town in the southwest of Nanjing, which is a new-built central business district in recent years. Driven by mega-events mentioned before, large-scale infrastructure investment is injected to transform a marginal space into a new town rapidly. With the characteristics of the high starting point of the new construction, convenient facilities and high living quality, this district has become the hot pot of the housing selection among the new-rich stratum in Nanjing. 

In fact, after the reform of the housing system in 1998, the traditional residential unit system mode under a planned economy was dissolved, and the newly-built gated housing became the mainstream in the living space for citizens in urban China. Under the influence of marketization, residents can freely choose location and type of housing in the housing market and the value of real estates is activated. Studies have shown that the positive externalities of green spaces will drive the value of real estates in surrounding areas [28,54,55,56], which is 8% to 20% in the United States [54] and 8.6% in Guangzhou [55]. In this situation, most residents have the same preference for green space but only the privileged family has greater spending power. Therefore, the conflict between urban residents’ demand for access to parks and their ability to afford it generates social segregation on the access to urban parks among different socio-economic statuses: As the rich keep congregating in the community with better green space service where the less-advantaged people could not afford. 

**Figure 5:**
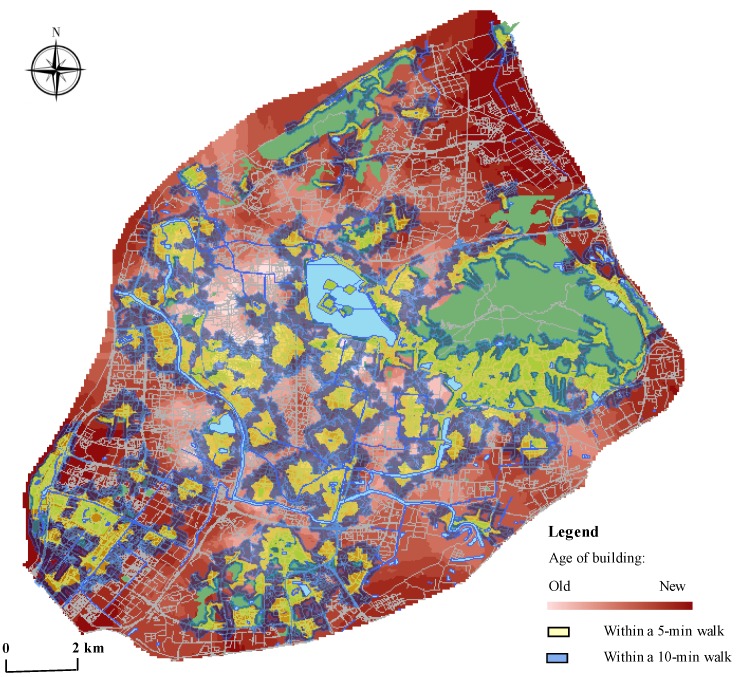
The distribution of park accessibility and the year-built of housings in Nanjing.

Figure 5 shows the accessibility and housing built–year distribution in Nanjing. The distribution pattern indicates that the housing age of Nanjing is decreasing from the center to the periphery. Also, most of the old communities were concentrated in the inner city of Nanjing with high urban park access while many underserved neighborhoods are in the suburbs, as shown in the figure. One possible explanation for this outcome could be the continuity and accumulation of urban development based on historical factors. Nanjing, an ancient capital with a long history of city construction, has many green resources that belong to the Scenic and Historic Interest Area, such as Xuanwu Lake and Ming Palace Park. With the local municipal endeavors over the decades, these historical city parks have undergone multiple rounds of environmental improvements. These green areas provide qualified parks and green space services to surrounding residents and play an important role in the contemporary urban green space system. Furthermore, in the aspect of residential locations, the old town within the ancient city wall was a major concentration area for the current population and urban development before the 21st century. At that time before housing reform in 1998, institutions offered free housing as welfare for their employees and the social stratification in the cities was not reflected in the living space. The social system of equalization results in the fairness of public resource allocation. Consequently, the majority of the dwellings in the inner city generally have evenly high accessibility to green parks. 

Nowadays, this planning legacy of China’s socialist era may still indirectly benefit marginalized residents. Some studies reveal that vulnerable groups, such as rural migrants and disadvantaged families, are clustered in the district with lower housing costs, primarily being consisted by decrepit settlements and dilapidated communities in the downtown [13,38,39]. Although these groups are not explicitly stated as the primary target groups, the location advantage of the old housing projects allows them to have access to urban parks in the inner city.

On the contrary, in the 21st century, the urban area in Nanjing expanded rapidly and a large number of commodity housing was quickly built. The development in the suburbs with car-oriented road networks and homogeneous land use is characterized by high strength, fast speed, and large scale. Pursuing the rapid pace of development and high productivity, the provision of urban public service facilities is relatively lagging behind, especially for high-quality urban parks. As shown in the Figure 5, for example, the number of urban parks is relatively scarce and the coverage of green space services is insufficient in the northern district of the city, including Baota Street and Xiaoshi Street in Gulou District, Maixuqiao Street and Yanziji Street in Qixia District, and some areas in the northwest of Hexi district. As a result, urban park service in inner-city old neighborhoods is on the whole much better than the newly-built districts in the suburb, except for some wealthy communities mentioned before.

### 4.4. Accessibility to Parks among the Vulnerable Groups

In order to further explore the correlation between housing prices, age of the building, and accessibility, these communities are divided into five partitions of equal size according to the housing price using the quinquepartite method of income in China [57]. Then, housing prices in the top 20%, 20% to 80%, and the bottom 20% could respectively represent high income, middle income, and low income, since housing attainment through the market depends to a large extent on disposable personal income [58]. The age of the building is divided into three sections, comprising before the 1998 housing reform, 1998 to 2008, and 2009 to the present. We summary the ratio of accessibility in a 10-min walk. 

Results from the chi-square tests in SPSS reveal statistically significant trends among the income groups (Table 5), indicating that affluent and middle-class residents tend to have a higher percentage of green spaces within the 10-min walking distance than low-income neighborhoods. Furthermore, the newly-built low-income communities have the lowest percentage of accessible green space, which is only half of the median of the overall accessibility to urban parks in Nanjing. 

There are mainly two types of residents living in the newly-built low-income communities, including the laid-off workers moving from the inner city and new migrants from the rural areas [13,38,39]. In the inner city, urban redevelopment with greening has become a filtering mechanism for urban living space transformation, causing the disadvantaged urban residents to move from the inner city and to the newly-built low-income communities in the suburb. For the sake of the rental gap, the government, investors and property developers choose the inner space with rich green space resources to launch the process of urban renewal. This renewal process takes urban green space as the engine to meet the preference of the middle class, leading to the increase of the property value and rents and the exclusion of the disadvantaged residents, being priced out by the sky-rocketing housing cost. Several authors define this process in which the improvement in urban parks has fueled the displacement of local residents as green gentrification [59,60,61]. 

For instance, in 2002, the urban river restoration projects of the Qinhuai River were launched in Nanjing to address the water pollution problem. By the end of 2009, the project had achieved a total investment of more than $60 million, involving several sub-projects such as water conservancy, environmental protection, housing settlements, and landscape construction. Due to these projects, the Qinhuai River was transformed into a new green belt with a series of riverside parks. However, the waterfront space has become the hot spot of private investors after the river pollution problem was solved, with its beautiful natural scenery and profound cultural atmosphere. High-end gated communities gradually appear around the Qinhuai River. Meanwhile, the traditional houses in the waterfront area, often lived by laid-off workers, were demolished in the process of river renewal. Unfortunately, the government compensation for these local low-income residents was far from enough to repurchase the newly-built apartments, which led to the relocation and displacement of these vulnerable residents. According to official statistics, more than 6000 residents in the area around the Qinhuai River were excluded and resettled to low-income communities in the periphery of the city during this redevelopment process. These high-quality urban parks and green space services are captured by residents of newly-built privileged communities, while indigenous disadvantaged residents are selectively filtered out in the process of green gentrification. As a result, to increase green space access with displacing or excluding underserved communities gives rise to environmental inequality.

Moreover, it is difficult for rural immigrants to purchase a housing unit through the real estate market after the reform of the housing commercialization reform facing rising housing costs and deteriorated housing affordability. Therefore, affordable housing has become one of the most salient urban social programs in the national policy agenda since 2007. The city governments support large-scale affordable housing construction in urban peripheral areas aimed to solve the housing problem of rural immigrants. As the common characteristics of these affordable housing projects, they are usually located in the outer area of the city, have relatively lower housing prices, and are usually constructed on a large scale. 

However, while the rapid development of affordable housing enabled the low-income groups to find their own living, major urban public service facilities, such as parks and green spaces, have not been distributed simultaneously. Consequently, it becomes inconvenient for vulnerable groups to receive the physical, mental, and social benefits afforded by park space, which exacerbates the trend of the unfair spatial distribution of urban green space. Our study illustrates that most of the affordable housing projects in Nanjing are located outside of the 10-min walking distance to the green space, revealing the lack of access to enjoy urban parks. For instance, located in the northeast corner of the central Nanjing city, Dingjiazhuang has a total of 3.1 million square meters of affordable housing. This affordable housing project fails to take the accessibility of urban parks, despite the fact that they do fulfill the housing demands of the newly-immigrated residents. 

## 5. Discussion

With the growing demand of urban residents for a better living environment, the benefits of green space as a type of public goods has received more and more attention [1,2,3,4,5,6]. Considerable studies indicate that similar to income inequality, housing inequality in urban China is increasing, and there is residential socio-spatial differentiation and that the choice of urban housing could reflect the socio-demographic status for the residents [17,18,19,20,21,22,23,24,25]. Furthermore, a question for urban sustainability planning is emerging about whether the unequal residential distribution of social groups would affect their access to public facilities, such as green space. In response to this issue, taking Nanjing as an example, we selected a 10-min walking distance as the residents’ threshold preference and calculated the accessibility of the green space at the house scale using ArcGIS street network analysis. Our research found this accessibility of urban parks is positively correlated with housing prices, and negatively correlated with the age of the buildings, holding all other factors constant. While affluent homeowners capture a high-quality green amenity, newly-built low-income communities, where most residents are vulnerable population, have the lowest percentage of accessible green space.

As a result of China’s transition to a market economy, the implementation of housing commodification and housing subsidy monetization policies has led to an active and dynamic real estate market in China. With the improvement of living standards, residents tend to pursue a high-quality living environment. Green space evolves into a rival commodity and captures the affluent residents with high affordability, which remodels the community atmosphere adjacent. The gap in access to green space among socio-economic groups has gradually widened. In addition, for the vulnerable groups, particularly for the urban poor and rural immigrants living in the dilapidated districts, are being displaced by the inner redevelopment in the form of “demolishing the old and constructing the new”. They were forced to move from the former living areas in the inner city to large-scale affordable houses in urban peripheral areas which lack public service facilities, such as parks and green spaces. This process of displacement and relocation exacerbates the trend of the unfair spatial distribution of urban green space and worsens the inequity among different socio-economic groups regarding their quality of life. 

### 5.1. Local Implication and Research Prospects

Although many government officials, planners, and private investors have carried out greening projects from the perspective of green cities and sustainable planning, little attention has been paid to the inequitable distribution of parks and green space in urban China. Most structured proposals are difficult to guarantee all resident’s benefits and their accessibility to the green space. Summarizing the urban master plan and sustainability plan formulated by Nanjing Municipality in the past 10 years, it was found that only 40% mentioned social equity, and only 10% of the work adopted a clear expression of environmental justice and its action points [41]. Therefore, green space planning should take both quantity and quality of urban parks into account within the current context of ecological civilization construction in urban China. To achieve the ideal outcome of the human-orientation design and to strengthen social equity, planners and local governments should consider the accessibility to parks and other public services as the main indicators for environmental equity. More specifically, future inner-city redevelopment projects and affordable housing projects should fully embed the supplement of public services and the amenities for different socio-economic groups in the planning process. This will help avoid the social segregation between the rich and poor, especially under the current transition of China’s economy. 

As the growing green gentrification researchers contend, many green interventions create environmental privileged enclaves, and exclude the low-incomes and marginal residents from these new green spaces [59,60,61]. In this process, some policy-makers even demonstrated clear strategies to attract commercial and residential investment and appeal to more residents with social and ethnic advantages [62]. Having more green spaces incorporated (like greenways, parks, and green infrastructure), these projects do not necessarily benefit people from all social stratums. For the reference of future practice, urban green interventions need to reconsider their impact on multiple scales and rebound the effects of environmental inequality brought by the urban green projects. Additionally, future research needs to further theorize and contextualize the issue of environmental justice on the global scale, especially in the Global South.

### 5.2. Strengths and Limitations

This article is the first quantitative examination of the green space accessibility among the different socio-economic groups within the context of residential differentiation in urban China. Our research provides a conceivable answer to the questions: Are the marginalized groups experiencing a lower degree of accessibility to urban green space in Nanjing, China, a typical metropolitan city? If yes, what are the reasons behind this inequality? This study provides a practical assessment and policy implications for other developing countries under similar settings, helping to mitigate the fact that public services are usually limited in the developing countries and that the distribution of service usually fails to be equitable, especially for the lower-income class. Lastly, in terms of methodology, our study used the house-level data from the secondary market in order to characterize residents’ access to local facilities more accurately with the assistance of the street network analysis. Our study also sheds light upon an innovative approach to address the lack of official micro-scale statistics and the lag in traditional data availability for contemporary urban studies in China. 

Besides the research findings and the discoveries revealed, this study has some limitations where further study needs to elaborate upon. First, we selected walking as the only mode of transportation to access the parks, further studies should consider controlling for other modes of transportation, such as bike, bus, metro, and private automobiles. Second, we chose to focus on the proximity of parks, and we suggest that other factors, like the specific acreage, function, quality, and other parameters of the individual park, should also be taken into account in future studies. Last, we used socio-economic status as the indicator to evaluate spatial equity. In the future study, the vulnerable groups could be further defined by their inadequate access to private recreations, including the youth, senior citizens, and the handicapped population. These measurements could contribute to a more accurate outcome regarding the assessment of accessibility.

## 6. Conclusions

We chose Nanjing as the empirical case in urban China and calculated the accessibility of the green space at the house scale with ArcGIS street network analysis. Our results show that 60.45% of the real estates in the main urban area of Nanjing are located within a 10-min walk to urban green space, indicating that most residents can benefit from the urban park service. Moreover, in terms of spatial equity, this study found that there is a significant positive correlation between the housing price and park accessibility. That is, households with a higher level of housing values have better access to urban parks. The age of the building has a significant negative correlation with the park accessibility, revealing that neighborhoods built in earlier years have better urban park accesses compared with the recent developments. The access of green space for newly constructed low-income communities is much lower than the median value of the overall park accessibility in Nanjing, especially after the reform of the housing system in 1998, indicating that disadvantaged residents were starting to be deviated from the urban parks in recent housing developments. Overall, under the background of rapid urbanization and the improvement of the quality of urban space in Nanjing, residents with higher socio-economic status enjoy better access to urban park green space services while the lower socio-economic groups could not. Also, our study highlights that there are existing spatial disparities of urban park access among the multiple social groups in Nanjing. Therefore, there should be an evolution from improving the urban green coverage to promoting the proper match of the distribution of urban public parks regarding the need among different socio-economic groups to achieve equitable and sustainable development. 

## Figures and Tables

**Table 1:** Descriptive statistics.

	Minimum	Maximum	Mean	Std. Deviation
Housing Price (USD/m^2^)	963	19,973	5099	12,981.94
Age of Building	73	0	21	10.44
HOA Fee (USD/m^2^)	0	1.29	0.13	0.90
Community Size	1	166	10	11.14

N = 2609.

**Table 2:** Reference standards of time-cost accessibility levels.

Accessibility Level	Time Cost	Distance	Valuation
Very Good	<5 min	<0.25 mi	4
Good	5–10 min	0.25–0.5 mi	3
Poor	10–15 min	0.25–0.75 mi	2
Very Poor	>15 min	>0.75 mi	1

**Table 3:** Statistics results of accessibility in Nanjing.

	District	Total	Inner-City	Suburb
Accessibility level	Sum	2609	1227	1382
Very good access (<5 min)	Number	473	277	196
	Percentage	18.13%	22.58%	14.18%
Good access (5~10 min)	Number	1104	611	493
	Percentage	42.32%	49.80%	35.67%
Poor access (10~15 min)	Number	754	339	415
	Percentage	28.90%	27.63%	30.03%
Very poor (>15 min)	Number	278	0	278
	Percentage	10.66%	0.00%	20.12%
Within a 10-min walk	Number	1577	888	689
	Percentage	60.44%	72.37%	49.86%

**Table 4:** Correlation between accessibility and housing attributes in Nanjing.

Housing Attributes	Analysis Method	N	Correlation Coefficient
Housing Price	Spearman rank correlation	2609	0.127 **
	Kendall rank correlation	2609	0.097 **
Age of the building	Spearman rank correlation	2609	−0.160 **
	Kendall rank correlation	2609	−0.123 **
HOA Fee	Spearman rank correlation	2292	−0.013
	Kendall rank correlation	2292	−0.010
Community Size	Spearman rank correlation	2609	−0.089
	Kendall rank correlation	2609	−0.069

** Correlation is significant at the 0.01 level.

**Table 5:** Ratio of accessibility in a 10-min walk of different housing attributes in Nanjing.

Year of Built	Low Income	Middle Income	High Income	Average	Pearson Chi-Square
Before 1998	51.16%	66.56%	80.42%	65.67%	51.573 **
1999–2008	34.00%	60.46%	67.27%	56.89%	41.802 **
After 2009	33.80%	48.67%	49.57%	46.36%	5.674 *
Average	43.87%	63.00%	69.35%	60.44%	81.575 **

* Indicates *p*-value < 0.05. ** Indicates *p*-value < 0.001.
